# Extracellular polysaccharides produced by *Ganoderma formosanum* stimulate macrophage activation via multiple pattern-recognition receptors

**DOI:** 10.1186/1472-6882-12-119

**Published:** 2012-08-10

**Authors:** Cheng-Li Wang, Chiu-Ying Lu, Chia-Chen Pi, Yu-Jing Zhuang, Ching-Liang Chu, Wen-Hsiung Liu, Chun-Jen Chen

**Affiliations:** 1 Department of Biochemical Science and Technology, National Taiwan University, Taipei, Taiwan, 10617, Republic of China; 2 Graduate Institute of Immunology, National Taiwan University, College of Medicine, Taipei, Taiwan, 100, Republic of China

**Keywords:** *Ganoderma formosanum*, Polysaccharide, Immunostimulatory, Macrophage, Pattern-recognition receptor

## Abstract

**Background:**

The fungus of *Ganoderma* is a traditional medicine in Asia with a variety of pharmacological functions including anti-cancer activities. We have purified an extracellular heteropolysaccharide fraction, PS-F2, from the submerged mycelia culture of *G. formosanum* and shown that PS-F2 exhibits immunostimulatory activities. In this study, we investigated the molecular mechanisms of immunostimulation by PS-F2.

**Results:**

PS-F2-stimulated TNF-α production in macrophages was significantly reduced in the presence of blocking antibodies for Dectin-1 and complement receptor 3 (CR3), laminarin, or piceatannol (a spleen tyrosine kinase inhibitor), suggesting that PS-F2 recognition by macrophages is mediated by Dectin-1 and CR3 receptors. In addition, the stimulatory effect of PS-F2 was attenuated in the bone marrow-derived macrophages from C3H/HeJ mice which lack functional Toll-like receptor 4 (TLR4). PS-F2 stimulation triggered the phosphorylation of mitogen-activated protein kinases JNK, p38, and ERK, as well as the nuclear translocation of NF-κB, which all played essential roles in activating TNF-α expression.

**Conclusions:**

Our results indicate that the extracellular polysaccharides produced by *G. formosanum* stimulate macrophages via the engagement of multiple pattern-recognition receptors including Dectin-1, CR3 and TLR4, resulting in the activation of Syk, JNK, p38, ERK, and NK-κB and the production of TNF-α.

## Background

In Asian traditional medicine, the fungus of *Ganoderma* (also called Reishi or Ling-Zhi) has been used, for thousands of years, as a health promoting supplement to treat various diseases [1], but not until recently have the pharmacologically active components in *Ganoderma* been purified and characterized [2,3]. Various pharmacologically active substances, including polysaccharides, triterpenoids, alkaloids, steroids, amino acids, proteins, nucleosides, and nucleotides have been isolated from *Ganoderma*[2-4]. The polysaccharide, protein, and triterpenoid components of *Ganoderma* have anti-tumor properties, which may function via their immunomodulatory activities [5]. Among the bioactive components, polysaccharides extracted from the fruiting bodies, or mycelia, of *Ganoderma* exhibit immunostimulatory activities on dendritic cells [6,7], monocytes/macrophages [8-10], neutrophils [11,12], and NK cells [13].

The innate immune system serves as the first line of defense against microbial infection, and functions primarily via the recognition of conserved microbial structures (the pathogen-associated molecular patterns or PAMPs) by pattern recognition receptors (PRRs) expressed on innate immune cells such as macrophages, neutrophils, and dendritic cells [14]. Among various PRRs identified to date, Toll-like receptors (TLRs) are the most well-characterized. Thirteen TLRs have been identified in humans and mice and each of which is specific for different PAMPs. TLRs are type I transmembrane proteins which have conserved N-terminal leucine-rich repeats and a cytoplasmic Toll/IL-IL-1R homology (TIR) domain. Upon activation by respective PAMPs, TLRs recruit a set of TIR domain-containing adaptor molecules and initiate signaling cascades that lead to the activation of NF-κB and IRFs and the expression of proinflammatory cytokines, chemokines, and type I interferons [15]. Many PAMPs are exposed and structurally conserved microbial surface structures, such as the outer membrane lipopolysaccharides (LPS) and cell wall peptidoglycan of bacteria, and components of the fungal cell wall. Gram-negative bacterial LPS is delivered to TLR4 via the accessory proteins LBP, CD14 and MD-2, and the activated TLR4 recruits four adaptor molecules: TIRAP, MyD88, TRAM, and TRIF. TLR4 interacts with TIRAP and MyD88 at the plasma membrane, and MyD88 further recruits IRAKs, TRAF6, and the TAK1 complex, resulting in the activation of NF-κB and mitogen-activated protein (MAP) kinases. At a later stage, TLR4 is endocytosed and delivered to intracellular vesicles, where it forms a complex with TRAM and TRIF, leading to IRF3 activation and the late-phase activation of NF-κB and MAPKs [15].

The fungal cell wall is predominantly composed of glycoprotein’s and carbohydrate polymers, including β-glucan, chitin and mannan, and, in most yeasts and molds, the cell wall polysaccharides have a core skeleton composed of branched β-1,3-glucans [16]. These cell wall components may serve as PAMPs and be recognized by a variety of host PRRs. TLR4 recognizes mannans expressed by *Saccharomyces cerevisiae* and *Candida albicans*[17]. Several receptors recognize β-glucan, including the C-type lectin receptor Dectin-1 [18], complement receptor 3 (CR3) [19], scavenger receptors [20], lactosylceramide [21], TLR2 [22], and TLR4 [6,9]. Of these Dectin-1 plays a major role in β-glucan recognition and control of fungal infection [23]. Activation of Dectin-1 by β-glucan leads to the initiation of spleen tyrosine kinase (Syk)- and caspase recruitment domain family member 9 (CARD9)-dependent signaling cascades, resulting in phagocytosis, respiratory burst, the activation of NF-κB and NFAT, and the expression of pro-inflammatory cytokines [24]. Dectin-1 can recognize the cell wall polysaccharides of various fungal species, including *Saccharomyces cerevisiae, Candida albicans,  Coccidiodes posadasii, Pneumocystis carinii, Aspergillus fumigatus*, and *Ganoderma lucidum *[25,26]. CR3 (Mac-1, CD11b/CD18) was the first receptor shown to recognize β-glucan via a distinct lectin domain [19,27,28]. CR3 activation by β-glucan triggers a downstream signaling involving Syk and phosphatidylinositol 3-kinase, leading to enhanced phagocyte killing of iC3b-opsonized tumor cells [29].

*Ganoderma formosanum* is a native species of *Ganoderma*, first isolated in Taiwan two decades ago. We previously established a submerged mycelia culture system of *G. formosanum* and purified the extracellular polysaccharides in the culture broth. The polysaccharides are mainly composed of D-mannose, D-galactose and D-glucose, and we showed that the major polysaccharide fraction PS-F2 could stimulate the activation of macrophages and protect mice against *Listeria monocytogenes* infection [30]. In this study, we further investigate the molecular mechanism of macrophage activation by PS-F2, and our results demonstrate that PS-F2 recognition is mediated by Dectin-1, CR3 and TLR4 on macrophages, leading to the activation of multiple signaling cascades involving Syk, JNK, p38, ERK and NK-κB in macrophages.

## Results and discussion

### Role of Dectin-1 and CR3 in PS-F2 stimulation

**Figure 1:**
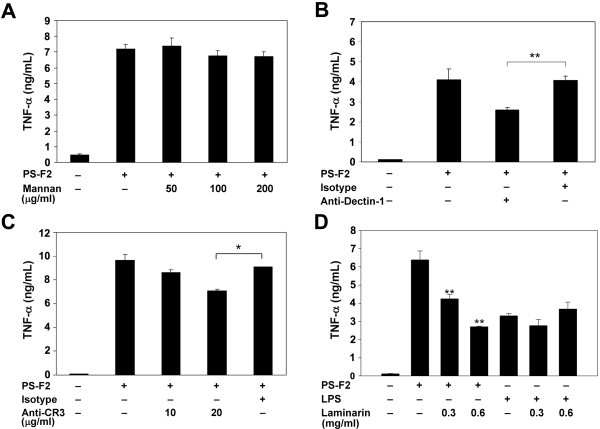
**PS-F2 stimulates macrophage activation via Dectin-1 and CR3-mediated pathways.** RAW264.7 cells were pre-incubated with or without mannan (**A**), anti-Dectin-1 antibody (2 μg/ml) (**B**), anti-CR3 antibody (**C**), or laminarin (**D**) for 1 h and stimulated with PS-F2 (10 μg/ml) or LPS (0.2 μg/ml) in the presence or absence of various reagents as indicated for additional 20 h. Cells left untreated, treated with isotype rat IgG2a antibody (**B**), or isotype rat IgG2b antibody (**C**) served as controls. TNF-α concentrations in the culture fluids were determined by ELISA (*n* = 3). Data shown are representative of 3 or more experiments. * *P <* 0.05, **P < 0.01 versus PS-F2 stimulation alone in (**D**).

To elucidate the mechanisms of PS-F2-stimulated macrophage activation, we first investigated what receptor(s) on macrophages could mediate the recognition of PS-F2. We hypothesized that PS-F2 functions as a fungal PAMP and interacts with certain sugar-binding PRR(s) on macrophages. C-type lectin receptors (CLRs) expressed on myeloid cells can recognize the carbohydrate structures on microorganisms [31]. Since mannose is the major carbohydrate component in PS-F2 [30], it is possible that a CLR with mannose specificity may mediate this interaction. However, the addition of mannan to the culture did not affect PS-F2-induced macrophage activation (Figure 1A), indicating that the PS-F2-binding receptor(s) on macrophages may not recognize the mannose moieties in PS-F2. Dectin-1 is a member of group V CLRs and serves as a PRR to sense the β-glucan in fungal cell wall [18]. To determine if Dectin-1 plays a role in PS-F2 recognition, RAW264.7 cells were stimulated with PS-F2 or zymosan in the presence of anti-Dectin-1 blocking antibodies. As expected, the stimulatory activity of zymosan was blocked by the antibodies (see Additional file 1). Anti-Dectin-1 antibodies also significantly suppressed PS-F2-stimulated TNF-α production (Figure 1B), indicating that PS-F2 stimulates macrophages, in part, via Dectin-1. It is likely that Dectin-1 recognizes β-glucan in PS-F2. However, since ligands other than β-glucan may also bind Dectin-1 [32], it remains possible that Dectin-1 may interact with other unique polysaccharide structures in PS-F2. 

CR3 is another receptor which recognizes fungal β-glucan [19], and the interaction is via a cation-independent lectin site located C-terminal to the I-domain of CD11b, which can be blocked by anti-CD11b mAb M1/70 [28]. Indeed, we found that this mAb was able to block zymosan-stimulated TNF-α production in macrophages (see Additional file 1). To determine if CR3 is involved in the recognition of the *G. formosanum* polysaccharides, PS-F2 stimulation was performed in the presence of M1/70 blocking antibodies for CR3. Results showed that TNF-α production was blocked by the anti-CR3 antibody in a dose-dependent way (Figure 1C). Dectin-1 and CR3 are, therefore, both involved in recognition of PS-F2. Laminarin is the β-glucan from brown algae and non-stimulatory, but blocks the stimulatory effects of various fungal β-glucans [33]. When stimulation was performed in the presence of laminarin, results showed that laminarin markedly and specifically inhibited PS-F2-induced macrophage activation (Figure 1D); in contrast, laminarin had no effect on LPS stimulation. The strong inhibition of PS-F2 stimulation by laminarin suggests that PS-F2 interaction with certain β-glucan-binding receptor(s) is responsible for macrophage activation. We have tried to determine whether the stimulatory function of PS-F2 is provided by β-glucan by treating PS-F2 with laminarinase; however the laminarinase of commercial source contained certain contaminants which also stimulated the activation of macrophages. Overall, these data suggest that the β-glucan receptors Dectin-1 and CR3 both play important roles in the recognition of PS-F2.

### Role of TLR4 in PS-F2 stimulation

**Figure 2:**
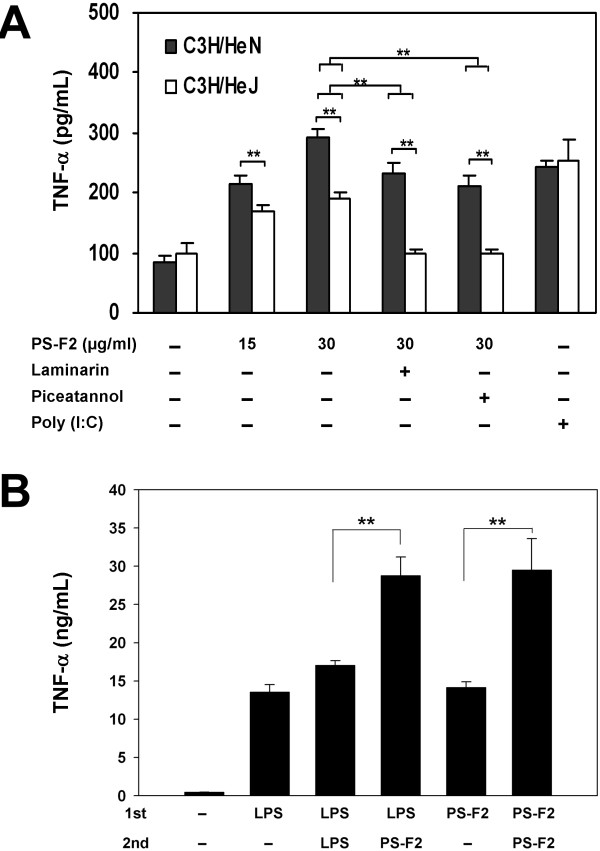
**The role of TLR4 in PS-F2-induced macrophage activation.** (**A**) BMDMs from C3H/HeN (wild type) and C3H/HeJ (TLR4 mutant) mice were pre-incubated with or without laminarin (0.3 mg/ml) or piceatannol (25 μM) for 1 h and stimulated with PS-F2 in the presence or absence of laminarin or piceatannol for 20 h. BMDMs left untreated or stimulated with poly (I:C) (10 μg/ml) served as controls. TNF-α concentrations in the culture fluids were determined by ELISA (*n* = 3). (**B**) RAW264.7 cells were stimulated with LPS (0.5 μg/ml) or PS-F2 (15 μg/ml) for 5 h (first stimulation), and after medium removal and washing, fresh media containing LPS (0.5 μg/ml) or PS-F2 (15 μg/ml) were added as indicated (second stimulation). At 20 h after second stimulation, TNF-α concentrations in the culture fluids were determined by ELISA (*n* = 3). Data shown are representative of 3 or more experiments. **P < 0.01.

Previous studies report that, in addition to Dectin-1 and CR3, TLR4 also recognizes fungal β-glucans, in particular the polysaccharides extracted from *G. lucidum *[6,9]. Although the polysaccharides purified from the submerged culture of *G. formosanum* appear distinct in sugar compositions from the polysaccharides extracted from *G. lucidum*[30,34], we also examined whether TLR4 plays a role in PS-F2 recognition. C3H/HeJ mice have a spontaneous mutation in the TLR4 gene and are thus resistant to endotoxin [35]. Upon PS-F2 stimulation, BMDMs from C3H/HeJ mice produced a significantly lower level of TNF-α compared with the BMDMs from wild-type C3H/HeN mice (Figure 2A). In contrast, the BMDMs from these two mouse strains showed similar responses to poly (I:C) (a TLR3 ligand) stimulation, indicating that PS-F2 specifically stimulates macrophages via TLR4. Consistent with the results in Figure 1D, addition of laminarin could suppress PS-F2-stimulated TNF-α production in both wild-type and TLR4-mutant BMDMs, and the stimulatory effect was almost completely eliminated in TLR4-mutant BMDMs when laminarin was present (Figure 2A). Although TLR2 has been reported to recognize fungal polysaccharides [22,36], it is not responsible for recognizing PS-F2 since BMDMs derived from wild-type and TLR2^−/−^ mice responded equally well to PS-F2 stimulation (see Additional file 2). Collectively, our data demonstrate that Dectin-1, CR3 and TLR4 are the three major receptors involved in the detection of PS-F2 by macrophages. Although the carbohydrate structure in PS-F2 that is recognized by TLR4 remains to be determined, it appears that TLR4 can detect carbohydrate-containing PAMPs. Several studies also report that polysaccharides from various fungal species, including *G. lucidum*, stimulate immune cell activation via TLR4 [6,9,36-38]. In addition, TLR4 also serves as a receptor for botanical polysaccharides which exhibit immunostimulatory activities [39]. Using a carbohydrate-receptor binding assay, a recent study showed that the polysaccharides extracted from *G. lucidum* interacted with a number of innate immune receptors, including Dectin-1, DC-SIGN, Langerin, Kupffer cell receptor, macrophage mannose receptor, TLR2 and TLR4 [40]. Based on our and others’ findings, it is clear that the innate immune cells can utilize multiple PRRs for recognition of the heteropolysaccharides in fungal cell walls. Different cell types may have different expression patterns of various PRRs, which would determine the outcome of polysaccharide stimulation. We have routinely observed that PS-F2 stimulated a significantly higher level of TNF-α production in RAW264.7 cells than in BMDMs. Besides the difference in cell origins (cell line vs. primary cell), we speculate that the relative expression levels of various PRRs may be different between these two types of macrophages, resulting in the difference in response to PS-F2 stimulation. 

Prior exposure of innate immune cells to LPS causes them to become refractory to subsequent LPS challenge, a phenomenon called “LPS tolerance” [41]. To test the possibility that prior LPS or PS-F2 exposure would make macrophages refractory to subsequent PS-F2 stimulation, RAW264.7 cells were stimulated with LPS or PS-F2, then subjected to secondary stimulation with LPS or PS-F2 5 hours later. As expected, LPS-exposed macrophages did not show further TNF-α production after second LPS challenge (Figure 2B). However, if cells were pretreated with LPS or PS-F2, subsequent PS-F2 stimulation could further increase the production of TNF-α (Figure 2B). These results indicate that, although TLR4 is one of the receptors for PS-F2, the “LPS tolerance” phenomenon does not occur upon PS-F2 stimulation, which may be due the activation of Dectin-1 and CR3. The data also excluded the possibility that the observed immunostimulatory activity of PS-F2 was caused primarily by LPS contamination in the samples.

### PS-F2-stimulated TNF-α production in macrophages requires the activation of MAPKs and NF-κB

**Figure 3:**
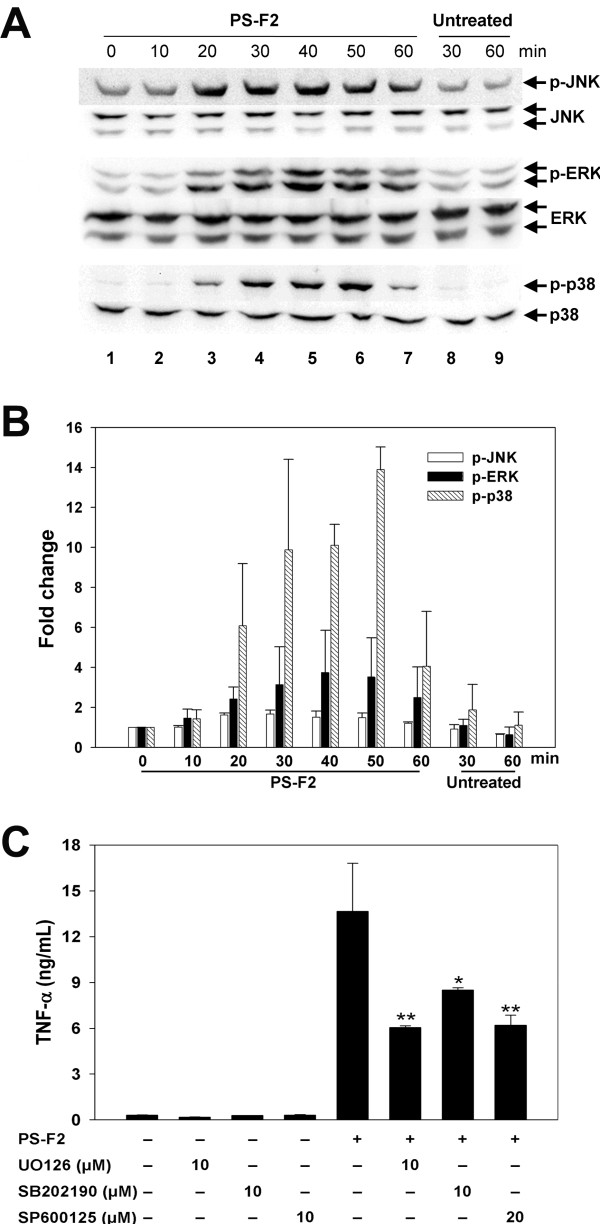
**Activation of MAPKs (JNK, ERK, and p38) is required for PS-F2-stimulated TNF-α production in macrophages.** (**A**) RAW264.7 cells were untreated or stimulated with PS-F2 (8 μg/ml), and total cell lysates were prepared at different times after stimulation. Equal amounts of cell lysates from the samples were subjected to Western blotting with antibodies specific for phosphorylated and total JNK, ERK and p38. (**B**) Signals in panel **A** were quantified by densitometric analysis, and phospho-specific signals were normalized against total protein signals. Bars indicate the fold changes of phosphorylated MAPK amounts at indicated time points over those at 0 min (*n* = 3). (**C**) RAW264.7 cells were pre-incubated with or without UO126 (ERK inhibitor), SB202190 (p38 inhibitor), or SP600125 (JNK inhibitor) for 30 min and stimulated with PS-F2 (15 μg/ml) for additional 20 h in the presence or absence of inhibitors. Cells left untreated or treated with inhibitors alone served as controls. TNF-α levels in the culture fluids were determined by ELISA (*n* = 3). Data shown are representative of 3 or more experiments. *P < 0.05, **P < 0.01 versus PS-F2 stimulation alone in (**C**).

**Figure 4:**
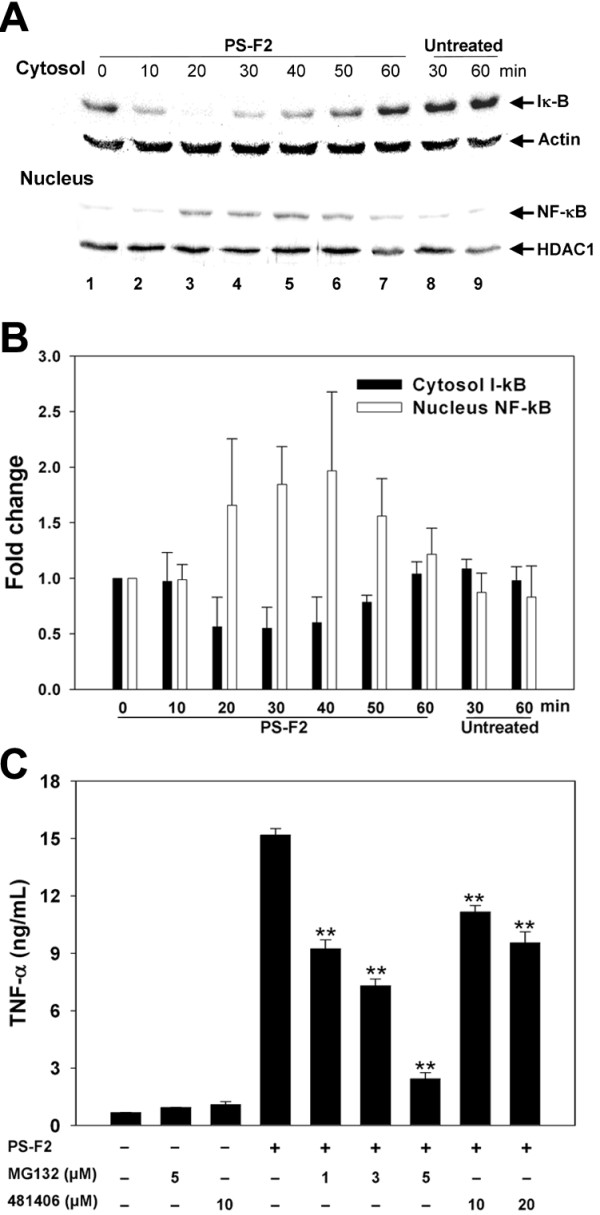
**NF-κB activation is required for PS-F2-stimulated TNF-α production in macrophages.** (**A**) RAW264.7 cells were untreated or stimulated with PS-F2 (8 μg/ml), and the cytosolic and nuclear fractions were prepared at indicated time points. Equal amounts of protein from the cytosolic fractions were subjected to Western blotting for I-κB and actin, and equal amounts of protein from the nuclear fractions were subjected to Western blotting for NF-κB and HDAC1. (**B**) Signals in panel A were quantified by densitometric analysis. I-κB signals were normalized against actin signals, and NF-κB signals were normalized against HDAC1 signals. Bars indicate the fold changes of I-κB and NF-κB amounts at indicated time points over those at 0 min (*n* = 3). (**C**) RAW264.7 cells were pre-incubated with or without MG132 or 481408 (NF-κB activation inhibitors) for 30 min and stimulated with PS-F2 (15 μg/ml) for additional 20 h in the presence or absence of inhibitors. Cells left untreated or treated with inhibitors alone served as controls. TNF-α levels in the culture fluids were determined by ELISA (*n* = 3). Data shown are representative of 3 or more experiments. **P < 0.01 versus PS-F2 stimulation alone in (**C**).

The MAPKs (ERK, JNK and p38) play critical roles in the downstream signaling of various PRRs including TLRs and Dectin-1 [42]. To characterize PS-F2-stimulated signaling pathways that lead to TNF-α production in RAW 264.7 cells, PS-F2 stimulation resulting in the phosphorylation and activation of MAPKs was first evaluated. Using antibodies specific for the phosphorylated JNK, p38 and ERK in Western blotting, protein phosphorylation was detected, starting at 20 min after PS-F2 stimulation (Figure 3A and 3B). To determine if activation of MAPKs plays a role in PS-F2-induced TNF-α production, RAW264.7 cells were stimulated with PS-F2 in the presence of MAPK inhibitors: UO126 (ERK inhibitor), SB202190 (p38 inhibitor), and SP600125 (JNK inhibitor). We have confirmed that theses inhibitors were effective in suppressing LPS-induced TNF-α production (see Additional file 3). As shown in Figure 3C, TNF-α production was significantly inhibited by U0126, SB202190, and SP600125, indicating that PS-F2-triggered activation of JNK, p38 and ERK all are involved in signaling for TNF-α production in RAW 264.7 cells. Besides MAPK signaling cascades, stimulation of various PRRs also leads to the degradation of I-κB by proteasome, which then allows NF-κB to translocate into the nucleus and activate the expression of proinflammatory cytokines. To determine whether PS-F2 stimulation could activate NF-κB, the levels of I-κB and NF-κB p65 subunit were assessed in the cytosolic and nuclear fractions, respectively. Upon PS-F2 stimulation, a transient (20–50 min), but clear, reduction of I-κB in the cytosol and a concomitant increase in NF-κB in the nucleus were noted (Figure 4A and 4B), indicating nuclear translocation and activation of NF-κB. We next determined whether the translocated NF-κB played a role in activating TNF-α expression by using the proteasome inhibitor MG132 and the NF-κB-specific inhibitor 481406. As a positive control, we found that both inhibitors effectively suppressed LPS-stimulated TNF-α production in RAW264.7 cells (see Additional file 3). When cells were treated with MG132 or 481406, PS-F2-stimulated TNF-α production was significantly reduced (Figure 4C). These results indicate that upon PS-F2 stimulation, both MAPK and NF-κB signaling pathways are activated and play important roles in the activation of TNF-α expression. 

### Syk mediates PS-F2-stimulated signaling and TNF-α production

**Figure 5:**
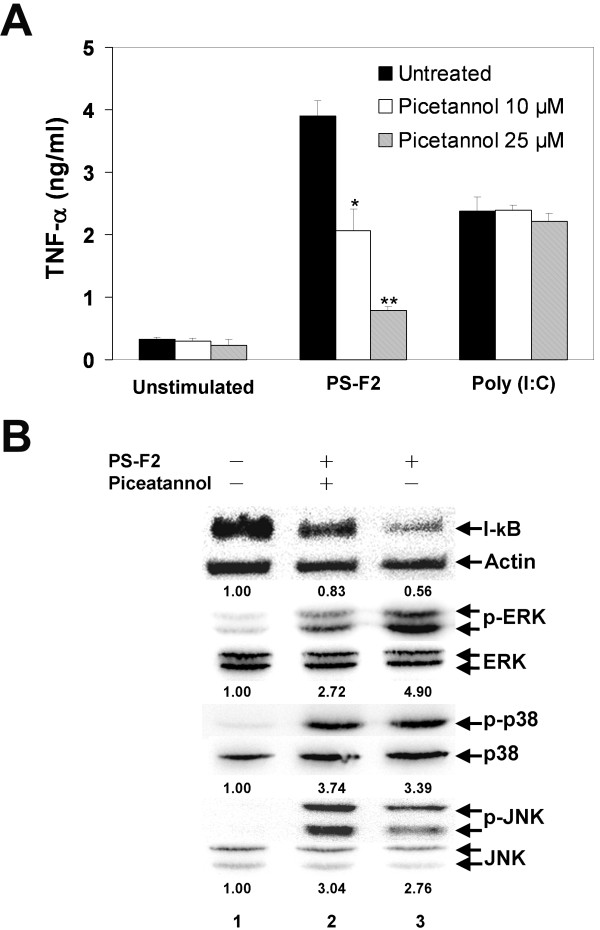
**Role of Syk activation in PS-F2-stimulated TNF-α production in macrophages.** (**A**) RAW264.7 cells were untreated or treated with piceatannol (10 and 25 μM) for 1 h and stimulated with PS-F2 (10 μg/ml) or poly (I:C) (10 μg/ml) for additional 20 h. Cells left unstimulated or stimulated with poly (I:C) served as controls. TNF-α concentrations in the culture fluids were determined by ELISA (n = 3). Data shown are representative of 3 or more experiments. (**B**) RAW264.7 cells were pre-incubated with or without piceatannol (25 μM) for 1 h and stimulated with PS-F2 (8 μg/ml) in the presence or absence of piceatannol. Cells left untreated served as controls. At 30 min after stimulation, cytosolic fractions and total cell lysates were prepared. Equal amounts of protein from the cytosolic fractions were subjected to Western blotting for I-κB and actin, and equal amounts of total cell lysates were subjected to Western blotting for phosphorylated and total JNK, ERK and p38. Changes in the I-κB signals (normalized against actin signals) and the phospho-specific signals (normalized against the total protein signals) are expressed as fold changes over the untreated group signals and indicated below each lane. * *P* < 0.05, **P < 0.01 versus PS-F2 stimulation alone in (**A**).

**Figure 6:**
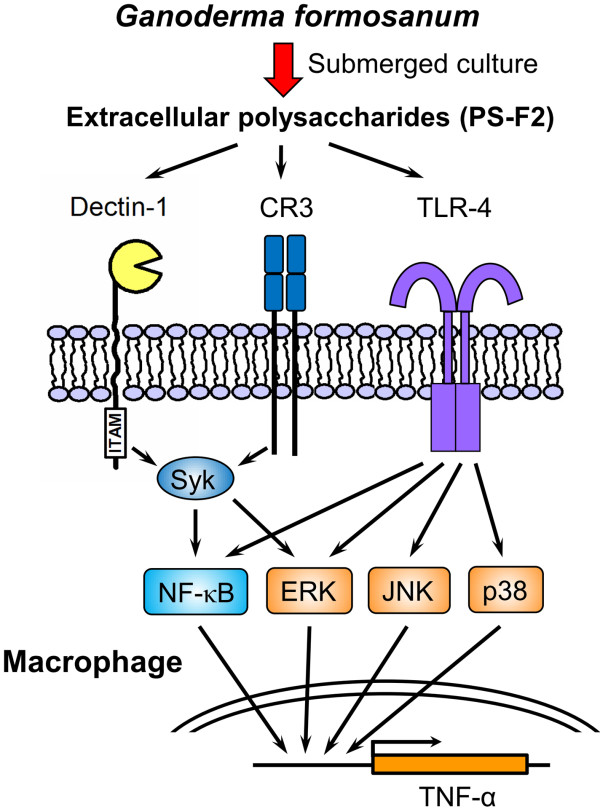
**Proposed mechanism for PS-F2-stimulated macrophage activation.** PS-F2 stimulates the activation of macrophage via the engagement of Dectin-1, CR3, and TLR4. The activation of these PRRs turned on the downstream signaling cascades involving Syk, JNK, p38, ERK and NK-κB, resulting in macrophage activation and TNF-α production.

Our data indicate that Dectin-1, CR3 and TLR4 could all serve as receptors for PS-F2. Syk kinase is a common signaling molecule downstream of Dectin-1 and CR3 [43], and we found that PS-F2-stimulated TNF-α production in macrophages was specifically and significantly suppressed by the Syk inhibitor piceatannol (Figure 5A and 2A). To further determine the contribution of Dectin-1, CR3 and TLR4 to downstream signaling, we examined whether the activation of MAPKs and NF-κB are regulated by Syk. Blocking Syk signaling by piceatannol prevented I-κB degradation and ERK phosphorylation but, in contrast, the phosphorylation of p38 and JNK was not affected (Figure 5B). These results indicate that, upon PS-F2 stimulation, Dectin-1 and CR3-mediated Syk activation leads to ERK phosphorylation and NF-κB activation, while TLR4 may contribute to the activation of p38, JNK, ERK and NF-κB. Similar to our observation, Syk signaling is important in zymosan-induced ERK activation in dendritic cells [42] (Figure 6). 

## Conclusion

In this study, we elucidate the molecular mechanism of macrophage activation by the heteropolysaccharide PS-F2 purified from the submerged culture of *G. formosanum*. Our data demonstrate that PS-F2 stimulates the activation of macrophage via the engagement of Dectin-1, CR3, and TLR4. The activation of these PRRs turned on the downstream signaling cascades involving Syk, JNK, p38, ERK and NK-κB, resulting in macrophage activation and TNF-α production. Together with the previous finding that PS-F2 could stimulate the activation of innate immune response in vivo and protect mice against *Listeria monocytogenes* infection [30], our results indicate that the extracellular polysaccharides of *G. formosanum* have the potential to be used as immunomodulatory agents in the treatment of infectious and malignant diseases.

## Methods

### Cell cultures and animals

Murine macrophage RAW264.7 cells were maintained as previously described [30]. Bone marrow-derived macrophages (BMDMs) were obtained by culturing bone marrow cells in DMEM (HyClone, UT, USA) supplemented with 10% fetal bovine serum (FBS) (Biological Industries, Beit-Haemek, Israel) and 30% L cell-conditioned medium for 7 days. C57BL/6 and C3H/HeN mice (6–8 weeks old) were purchased from the National Laboratory Animal Center (Taipei, Taiwan). C3H/HeJ (TLR4 mutant) mice were kindly provided by Dr. Zaodung Ling (National Health Research Institutes, Taiwan). TLR2^−/−^ mice were kindly provided by Dr. Shu-Mei Liang (Academia Sinica, Taiwan). All animal studies were approved by the Institute Animal Care and Use Committee of National Taiwan University, and all mice were kept in the animal facilities of the College of Life Science at National Taiwan University.

### PS-F2 and reagents

The major polysaccharide fraction PS-F2 was purified from the submerged culture of *G. formosanum* as previously described [30], and the endotoxin level was determined to be less than 0.3 EU/mg by the *Limulus* Amebocytes Lysate (LAL) test (Associates of Cape Cod Inc., East Falmouth, MA, USA). LPS (from *E. coli* O111:B4), laminarin, mannan, and polymyxin B were purchased from Sigma-Aldrich (St. Louis, MO, USA). SB202190, 481406, U0126, SP600125, and piceatannol were purchased from Calbiochem (Darmstadt, Germany). Poly (I:C) was purchased from InvivoGen (San Diego, CA, USA). Anti-CR3 mAb (M1/70), rat IgG2a and rat IgG2b isotype control antibodies were purchased from eBioscience (San Diego, CA, USA). Anti-Dectin-1 mAb (218820) was purchased from R&D Systems (Minneapolis, MN, USA). All other chemicals were purchased from commercial sources at the highest purity available.

### Cytokine production analysis

RAW264.7 cells grown in 96-well plates (1 × 10^5^ cells/well) were treated with polysaccharide samples, LPS or left untreated for 20 h, and mouse TNF-α levels in the culture medium were determined by ELISA (eBioscience). In some experiments, cells were pre-treated with various inhibitors or blocking antibodies for 30 min or 1 h, as indicated in the figure legends, prior to the addition of PS-F2.

### Preparation of cell lysates

To prepare whole cell lysates for MAPK phosphorylation analysis, RAW 264.7 cells plated in 6-cm dishes (2 × 10^6^ cells) were pre-incubated in serum-free DMEM for 2 h before stimulated with PS-F2 (8 μg/ml). At various time after stimulation, whole cell lysates were prepared by treating cells with 200 μl of SDS-PAGE sample buffer (62.5 mM Tris–HCl, 2% SDS, 20% glycerol, 10% 2-mercatoethanol, pH 6.8). To prepare cytoplasmic and nuclear extracts, cells were harvested and resuspended in 150 μl of hypotonic buffer (10 mM HEPES, pH 7.9, 10 mM KCl, 0.1 mM EDTA, 0.1 mM EGTA, 1 mM DTT, 0.5 mM PMSF) and incubated on ice for 15 min. The samples were then mixed with 10 μl of 10% NP-40 and centrifuged at 16,000 × g for 30 sec. The supernatant representing the cytosolic fraction was collected, and the pellet containing the nuclei was resuspended in 50 μl of nuclear extract buffer (20 mM HEPES, pH 7.9, 0.4 M NaCl, 1 mM EDTA, 0.1 mM EGTA, 1 mM DTT, 0.5 mM PMSF) and incubated at 4°C for 15 min with vigorous shaking. After centrifugation at 16,000 × g for 5 min, the supernatant representing the nuclear fraction was collected and stored at −20°C.

### Western blot analysis

Cell lysates in SDS-PAGE sample buffer were heated at 95°C for 5 min, separated by 12.5% SDS-PAGE, and transferred to a nitrocellulose membrane. The membrane was blocked with 5% bovine serum albumin in Tris-buffered saline containing 0.05% Tween 20 (TBST) and incubated with primary antibodies specific for JNK, p38, ERK, phospho-JNK, phospho-p38, phospho-ERK, NF-κB, I-κB (Cell Signaling, Danvers, MA, USA), β-actin (Santa Cruz Biotechnology, Santa Cruz, CA, USA), or histone deacetylase 1 (HDAC1) (Upstate Biotechnology, Waltham, MA, USA) at 4°C for overnight. The membrane was then incubated with horseradish peroxidase-conjugated secondary antibodies and visualized with an enhanced chemiluminescence kit (VisGlow, Taiwan) and a chemiluminescence imaging system (UVP Autochemi, Upland, CA, USA). Densitometric analysis of band intensities was performed using the ImageJ software (National Institutes of Health).

### Statistical analysis

Statistical analysis was performed using an unpaired, two-tailed Student's *t*-test and a *P* < 0.05 was considered significant. Data are reported as mean and SEM.

## Abbreviations

BMDM: Bone marrow-derived macrophages; CR3: Complement receptor 3; FBS: Fetal bovine serum; LPS: Lipopolysaccharides; MAPK: Mitogen-activated protein kinases; PAMP: Pathogen-associated molecular pattern; PBS: Phosphate-buffered saline; PRR: Pattern recognition receptors; SDS–PAGE: Sodium dodecyl sulphate–polyacrylamide gel electrophoresis; TLR: Toll-like receptor; TNF-α: Tumor necrosis factor-α.

## Competing interests

The authors declare that they have no competing interests.

## Authors’ contributions

CLW, CYL, CCP, YJZ, and CJC designed research. CLW, CYL, CCP, and YJZ performed research. CLC and WHL contributed new reagents/analytic tools. CLW and CJC wrote the paper. All authors read and approved the final version of the manuscript.

## Pre-publication history

The pre-publication history for this paper can be accessed here:

http://www.biomedcentral.com/1472-6882/12/119/prepub

## Supplementary Material

Additional file 1 Zymosan-stimulated macrophage activation was blocked by anti-Dectin-1 and anti-CR3 antibodies. Click here for file

Additional file 2 PS-F2-stimulated macrophage activation does not require TLR2. Click here for file

Additional file 3 LPS-stimulated macrophage activation was blocked by the inhibitors of MAPK and NF-κB activation. Click here for file
