# Supplementary material for: Extracellular polysaccharides produced by Ganoderma formosanum stimulate macrophage activation via multiple pattern-recognition receptors
Source: BMC Complement Altern Med. 2012 Aug 10;12:119. doi: 10.1186/1472-6882-12-119 (PMC3495220; doi:10.1186/1472-6882-12-119)
Supplement: Additional file 1 — Zymosan-stimulated macrophage activation was blocked by anti-Dectin-1 and anti-CR3 antibodies. [file 1472-6882-12-119-S1.pdf]

Additional file 1

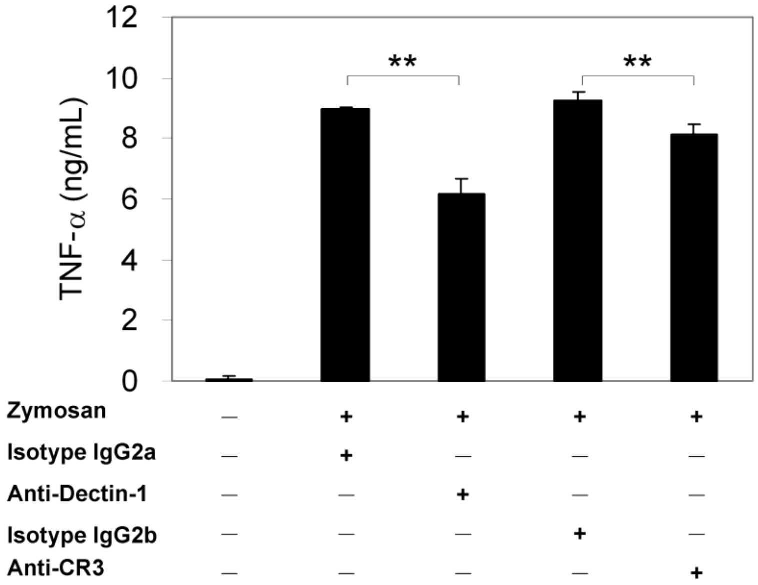

Zymosan-stimulated macrophage activation was blocked by anti-Dectin-1 and anti-CR3 antibodies. RAW264.7 cells were stimulated with zymosan (25 µg/ml) in the presence of anti-Dectin-1 antibody (2 µg/ml), anti-CR3 antibody (20 µg/ml), or same amounts of isotype control antibodies (rat IgG2a for anti-Dectin-1 antibody and rat IgG2b for anti-CR3 antibody). Cells left untreated served as the control. TNF-α concentrations in the culture fluids were determined by ELISA (n = 3). $^{**}P < 0.01$.
