# Supplementary material for: Extracellular polysaccharides produced by Ganoderma formosanum stimulate macrophage activation via multiple pattern-recognition receptors
Source: BMC Complement Altern Med. 2012 Aug 10;12:119. doi: 10.1186/1472-6882-12-119 (PMC3495220; doi:10.1186/1472-6882-12-119)
Supplement: Additional file 2 — PS-F2-stimulated macrophage activation does not require TLR2. [file 1472-6882-12-119-S2.pdf]

**Additional file 2**

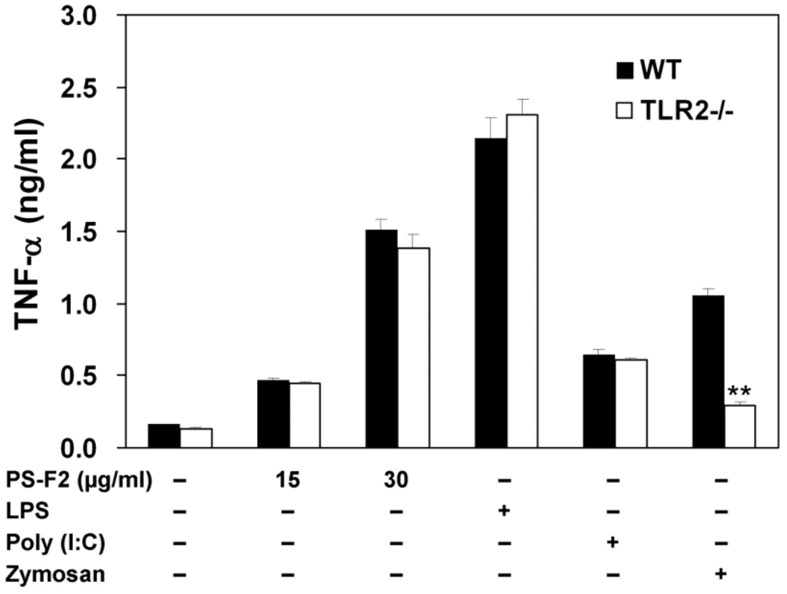


**PS-F2-stimulated macrophage activation does not require TLR2.** BMDMs from C57BL/6 (WT) and TLR2$^{-/-}$ mice were stimulated with PS-F2 (15 and 30 μg/ml). Cells left untreated, treated with LPS (0.5 μg/ml) or poly (I:C) (10 μg/ml) served as negative controls. Cells treated with zymosan (10 μg/ml) served as positive controls. At 24 h after treatment, TNF-α concentrations in the culture fluids were determined by ELISA ($n$ = 3). ** $P < 0.01$ versus WT.
