# Supplementary material for: Extracellular polysaccharides produced by Ganoderma formosanum stimulate macrophage activation via multiple pattern-recognition receptors
Source: BMC Complement Altern Med. 2012 Aug 10;12:119. doi: 10.1186/1472-6882-12-119 (PMC3495220; doi:10.1186/1472-6882-12-119)
Supplement: Additional file 3 — LPS-stimulated macrophage activation was blocked by the inhibitors of MAPK and NF-κB activation. [file 1472-6882-12-119-S3.pdf]

Additional file 3

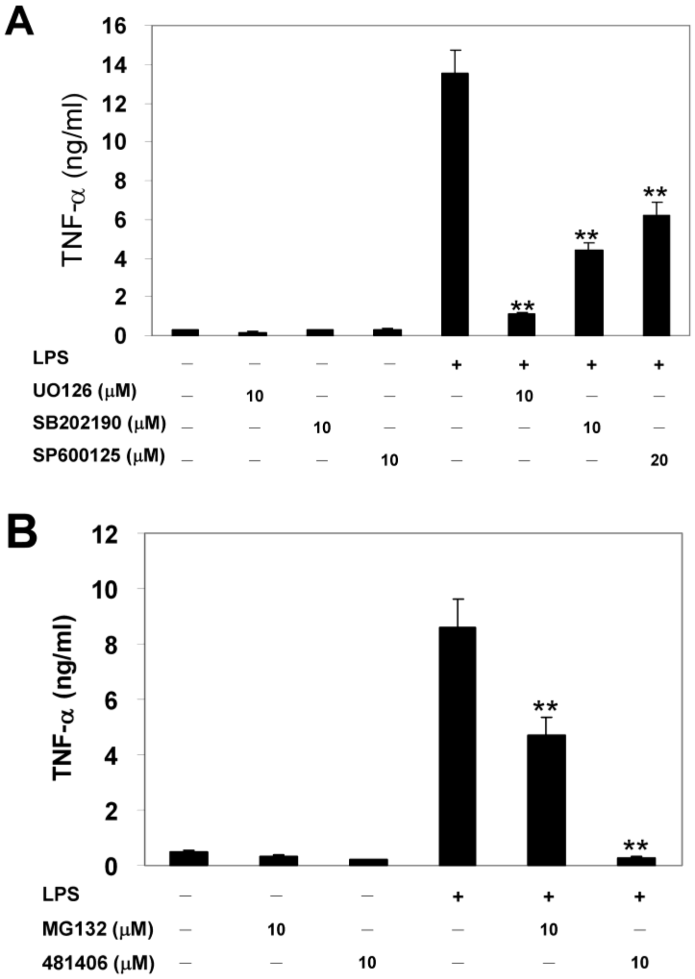

LPS-stimulated macrophage activation was blocked by the inhibitors of MAPK and NF-κB activation. (A) RAW264.7 cells were pre-incubated with or without UO126 (ERK inhibitor), SB202190 (p38 inhibitor), or SP600125 (JNK inhibitor) for 30 min and stimulated with LPS (1 μg/ml) for additional 20 h in the presence or absence of inhibitors. (B) RAW264.7 cells were pre-incubated with or without MG132 or 481408 (NF-κB activation inhibitors) for 30 min and stimulated with LPS (1 μg/ml) for additional 20 h in the presence or absence of inhibitors. In (A) and (B), cells left untreated or treated with inhibitors alone served as controls. TNF-α levels in the culture fluids were determined by ELISA (n = 3). **P < 0.01 versus LPS stimulation alone.
